# Supplementary material for: Diamagnetically levitated nanopositioners with large-range and multiple degrees of freedom
Source: Nat Commun. 2022 Jun 9;13:3334. doi: 10.1038/s41467-022-31046-4 (PMC9184538; doi:10.1038/s41467-022-31046-4)
Supplement: Supplementary file 1 — Supplementary Information [file 41467_2022_31046_MOESM1_ESM.pdf]

Supplementary information for:
Diamagnetically levitated nanopositioners with large-range and multiple degrees of freedom

K. S. Vikrant[1] & G. R. Jayanth[1,2*]

[1] Department of Instrumentation and Applied Physics, Indian Institute of Science, Bangalore, 560012. [2] Department of Mechanical Engineering, Indian Institute of Science, Bangalore, 560012. (Email: jayanth@iisc.ac.in)

Supplementary Note 1: Modeling and analysis of diamagnetic interaction

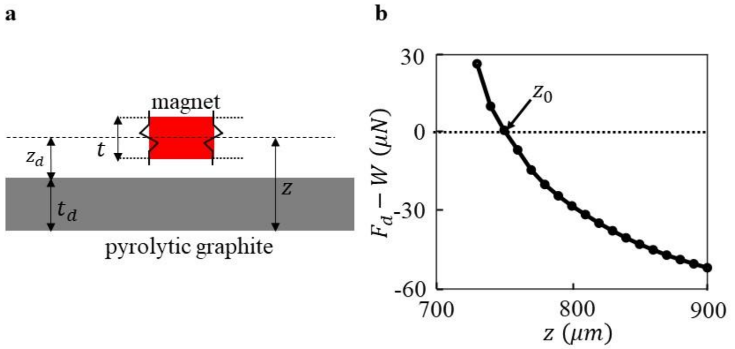

Supplementary Fig. 1. **Schematic used for calculating the diamagnetic force and the plot showing the dependence of calculated force on Z-position.** (a) A magnet of thickness t located at a height z above a pyrolytic graphite plate of thickness t_d,(b) Plot showing the net Z-force $F_d - W$ acting on the magnet as a function of the Z-position of the magnet. The dimensions of the magnet and the pyrolytic graphite plate used in the model are 1.7 mm × 1.7 mm × 0.4 mm and 3 mm × 3 mm × 0.5 mm respectively. The dimension of the enclosing air sphere is considered an order of magnitude greater than the length of the pyrolytic graphite plate. The magnetization and density of the magnet were considered as 10^6 *A/m* and *7200 kg/m³* respectively. The magnetic susceptibility of the pyrolytic graphite and the air were considered as -4.1 × 10^{-4} and 0 respectively.

Supplementary Fig. 1(a) shows the schematic of a magnet located at a height z above the lower face of a pyrolytic graphite plate of thickness t_d. To evaluate the dependence of force of diamagnetic repulsion between graphite and the magnet, finite-element method was employed, and was performed using COMSOL™ Multiphysics software. First a 3-D model comprising a single permanent magnet placed over a pyrolytic graphite plate and enclosed by an air sphere was developed. Subsequently the material properties required for the force calculation were specified, in particular, the magnetization of the permanent magnet and magnetic susceptibilities of graphite and air. Finally, appropriate boundary conditions were applied. The diamagnetic force F_d was calculated for 18 different Z-positions of the magnet starting from z = 900 μm and subsequently reducing the height by 20 μm till z = 730 μm. The plot in Supplementary Fig. 1(b) shows the net force $(F_d - W)$ acting on the permanent magnet a function of z, where W represents the weight

of the magnet. The levitation height z_0 is one at which the diamagnetic repulsion equals the weight of the magnet. The plot shows that the free levitation height z_0 is 752 µm.

Supplementary Note 2: Modeling and analysis of forces and torque on the actuator

Since the four traces are identical, the analysis of the forces on the magnet array is obtained in three steps. First, the magnetic field generated in the levitating plane by a single trace aligned along the Y-axis is obtained. Next, this is used to obtain the force and torque due to a single trace and subsequently, the force and torque due to all the four Y-traces are obtained. By expoloiting the symmetry in the arrangement of traces, the results are extended to traces along the X-axis.

2.1. Magnetic field due to a single Y-trace

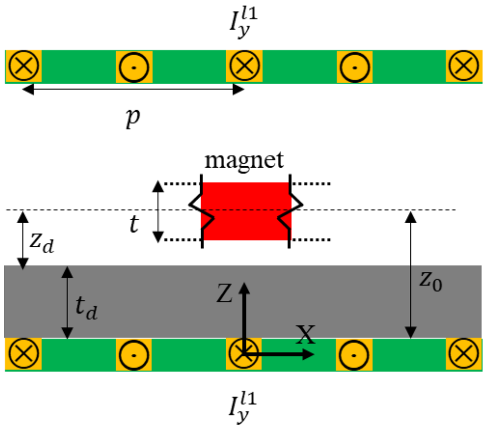

Supplementary Fig. 2. **Schematic of a magnet in the array situated between the top and bottom traces.** A cross-sectional view of the actuator with a single trace for purposes of modeling has been shown. In the schematic t and t_d represents the thickness of the magnet and the pyrolytic graphite plate respectively. Further, the separation gap between the levitation plane of the magnet and the PCB is represented by z_0. The distance between the center of the magnet and the top surface of the pyrolytic graphite plate is represented as z_d.

Supplementary Fig. 2 shows a schematic of a magnet in the magnet array positioned between the top and the bottom actuating traces. A single trace along the Y-axis comprises 2N straight conductors, with N of them carrying current along the positive Y-axis and the remaining N carrying current along the negative Y-axis. The magnetic field set up by the trace would possess components only in the X-Z plane and are obtained by adding the field due to each of these straight conductors. By symmetry, the same analysis can be employed to obtain the magnetic field generated by traces aligned along the X-axis.

In obtaining the magnetic field away from the edges of the traces, the conductors are assumed to be infinitely long. The X- and Z-components of the field $b_{kx}(x,z)$, $b_{kz}(x,z)$ of the kth conductor at a point (x,z) in the plane, obtained from Biot-Savart's law, are given by

$$b_{kz}(x,z) = (-1)^{k+1}\frac{\mu_0}{2\pi}\frac{x+\frac{kp}{2}}{\left[\left(x+\frac{kp}{2}\right)^2+z^2\right]} = b_0\tilde{b}_{kz}(\tilde{x},\tilde{z}) \;, \tag{1}$$

$$b_{kx}(x,z) = (-1)^{k}\frac{\mu_0}{2\pi}\frac{z}{\left[\left(x+\frac{kp}{2}\right)^2+z^2\right]} = b_0\tilde{b}_{kx}(\tilde{x},\tilde{z}). \tag{2}$$

where, $b_0 = \mu_0/2\pi p$, $\tilde{x} = x/p$ and $\tilde{z} = z/p$, while $\tilde{b}_{kz} = (-1)^{k+1}\frac{\tilde{x}+k/2}{[(\tilde{x}+k/2)^2+\tilde{z}^2]}$, and $\tilde{b}_{kx} = (-1)^k \frac{\tilde{z}}{[(\tilde{x}+k/2)^2+\tilde{z}^2]}$.

The total magnetic field per unit current $b_i(x,z), (i = x, z)$ set up by one trace is given by

$$b_i(x,z) = b_0 \sum_{k=-N}^{N} \tilde{b}_{ki}(\tilde{x},\tilde{z}) = b_0 \tilde{b}_i(\tilde{x},\tilde{z}), \quad (3)$$

where, $\tilde{b}_i = \sum_{k=-N}^{N} \tilde{b}_{ki}$.

In the limit that N is large, the magnetic field far away from the edges of the PCB can be approximated to be

$$\tilde{b}_i(\tilde{x},\tilde{z}) \approx \sum_{k=-\infty}^{\infty} \tilde{b}_{ki}(\tilde{x},\tilde{z}). \quad (4)$$

This field is periodic. It is also worth noting that

$$b_i\left(x + \frac{p}{2}, z\right) = -b_i(x,z). \quad (5)$$

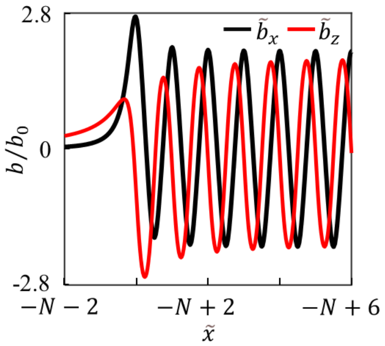

Supplementary Fig. 3. **Graph showing the variation of normalized X- and Z-magnetic fields with X-position:** Plots showing the normalized X-magnetic field $\tilde{b}_x$ and Z-magnetic field $\tilde{b}_z$ due to a single current-carrying track parallel to the Y-axis. The number of straight-line segments constituting the Y-track is considered as $2N = 100$ which starts from $\tilde{x} = -N$ and ends at $\tilde{x} = N$.

Supplementary Fig. 3 plots $\tilde{b}_x(x, z_0)$ and $\tilde{b}_z(x, z_0)$ and shows that they are nearly sinusoidal away from the edge of the trace. They also show that within just about one pitch of the trace, the magnetic fields converge to their sinusoidal profile. Thus, it is of particular interest to compute the first harmonic of this field at a height $z = z_0$. Since $b_x(x, z_0)$ is an even function of x, the first harmonic would be of the form $b_{1x} \cos\left(\frac{2\pi}{p}x\right)$, where b_{1x} is given by

$$b_{1x}(z_0) = \frac{2}{p}\int_{-\frac{p}{2}}^{\frac{p}{2}} b_x(x, z_0) \cos\left(\frac{2\pi}{p}x\right) dx. \quad (6)$$

Likewise, since $b_z(x, z_0)$ is an odd function of x, the first harmonic would be of the form $b_{1z} sin\left(\frac{2\pi}{p}x\right)$, where b_{1z} is given by

$$b_{1z}(z_0) = \frac{2}{p}\int_{-\frac{p}{2}}^{\frac{p}{2}} b_z(x, z_0) \sin\left(\frac{2\pi}{p}x\right) dx. \quad (7)$$

2.2. Dependence of magnetic stiffness and loads on the currents through the traces

The forces and torque generated by the four Y- traces are first analyzed. Since all the four Y-traces are identical, the loads applied by a single trace are considered first. Next, to obtain the overall load, these expressions are shifted by appropriate amounts along the X- and Z-axes and then added together. By symmetry, the same would be applicable for traces aligned along the X-axis.

The force $\mathbf{F_1}$ and the torque $\boldsymbol{\tau_1}$ on a single magnet with uniform magnetization $\mathbf{M}$ and volume V in a magnetic field $\mathbf{B}$ is given by

$$\mathbf{F_1} = \int_V \nabla(\mathbf{M}\cdot\mathbf{B})dV, \tag{8}$$

$$\boldsymbol{\tau_1} = \int_V \mathbf{M}\times\mathbf{B}dV. \tag{9}$$

The magnetization is aligned along the Z-axis. The magnetic field $\mathbf{B}^{\boldsymbol{\ell}\mathbf{1}}$ due to the first Y-trace on the lower PCB, designated by the superscript ℓ_1 would possess only X- and Z-components, i.e., $\mathbf{B}^{\boldsymbol{\ell}\mathbf{1}} = B_x^{\ell 1}\hat{\mathbf{x}} + B_z^{\ell 1}\hat{\mathbf{z}}$. Thus, the force $\mathbf{F}_1^{\ell 1}$ and the torque $\boldsymbol{\tau}_1^{\ell 1}$ in the levitation plane $z = z_0$ due to this trace alone are given by

$$\mathbf{F}_1^{\ell 1} = M\int_V \partial_x B_z^{\ell 1}(x,z_0)dV\,\hat{\mathbf{x}} + M\int_V \partial_z B_z^{\ell 1}(x,z_0)dV\,\hat{\mathbf{z}}, \tag{10}$$

$$\boldsymbol{\tau}_1^{\ell 1} = M\int_V B_x^{\ell 1}(x,z_0)dV\,\hat{\mathbf{y}}. \tag{11}$$

In Eqn. (10), ∂_i represents the operator $\partial/\partial i$. Next, it is noted that $B_i^{\ell 1}(x,z_0) = b_i(x,z_0)I_y^{\ell 1}$ $(i = x,z)$. Furthermore, since $b_i\left(x+\frac{p}{2},z\right) = -b_i(x,z)$, and the next neighboring magnetic moment is pointed in the opposite direction, the force and the torque experienced by this magnet would be identical to the first one. Consequently, the total force on an magnet array comprising N magnets due to a single trace is given to be

$$\mathbf{F}^{\ell 1} = N\mathbf{F}_1^{\ell 1}, \tag{12}$$

$$\boldsymbol{\tau}^{\ell 1} = N\boldsymbol{\tau}_1^{\ell 1}. \tag{13}$$

The X-component of this force is given by

$$F_x^{\ell 1} = NM\left[\int_V \partial_x b_z(x,z_0)\,dV\right]I_y^{\ell 1}. \tag{14}$$

Similarly, the X-component of the forces due to the other Y-traces are given by

$$F_x^{\ell 2} = NM\left[\int_V \partial_x b_z\left(x-\frac{p}{4},z_0\right)dV\right]I_y^{\ell 2}, \tag{15}$$

$$F_x^{u1} = NM\left[\int_V \partial_x b_z(x,-z_0)\,dV\right]I_y^{u1}\ , \tag{16}$$

$$F_x^{u2} = NM\left[\int_V \partial_x b_z\left(x-\frac{p}{4},-z_0\right)dV\right]I_y^{u2}. \tag{17}$$

Noting that since $\partial_x b_z(x,z)$ is an even function of z, it is concluded that $\partial_x b_z(x,-z_0) = \partial_x b_z(x,z_0)$. Thus, the forces due to the four conductors carrying currents $I_y^{\ell 1}, I_y^{u1}, I_y^{\ell 2}, I_y^{u2}$ are given to be

$$F_x = NM\left[\int_V \partial_x b_z(x,z_0)\,dV\right]\left(I_y^{\ell 1}+I_y^{u1}\right) + NM\left[\int_V \partial_x b_z\left(x-\frac{p}{4},z_0\right)dV\right]\left(I_y^{\ell 2}+I_y^{u2}\right). \tag{18}$$

Likewise, utilizing the fact that $\partial_z b_z(x,-z_0) = -\partial_z b_z(x,z_0)$, the Z-force due to the Y- trace F_{zy} is given by

$$F_{zy} = NM\left[\int_V \partial_z b_z(x,z_0)\,dV\right]\left(I_y^{\ell 1}-I_y^{u1}\right) + NM\left[\int_V \partial_z b_z\left(x-\frac{p}{4},z_0\right)dV\right]\left(I_y^{\ell 2}-I_y^{u2}\right). \tag{19}$$

Finally, utilizing the fact that $b_x(x, -z_0) = -b_x(x, z_0)$, the torque is given by

$$\tau_y = NM\left[\int_V b_x(x, z_0)\, dV\right]\left(I_y^{\ell 1} - I_y^{u1}\right) + NM\left[\int_V b_x\left(x - \frac{p}{4}, z_0\right) dV\right]\left(I_y^{\ell 2} - I_y^{u2}\right). \quad (20)$$

The stiffness along the X-axis is given by

$$k_x = -\frac{\partial F_x}{\partial x} = -NM\left[\int_V \partial_{xx} b_z(x, z_0)\, dV\right]\left(I_y^{\ell 1} + I_y^{u1}\right) - NM\left[\int_V \partial_{xx} b_z\left(x - \frac{p}{4}, z_0\right) dV\right]\left(I_y^{\ell 2} + I_y^{u2}\right). \quad (21)$$

In a similar manner, the forces F_y, F_{zx}, the torque τ_x and the stiffness k_y can be derived and are given to be

$$F_y = NM\left[\int_V \partial_y b_z(y, z_0)\, dV\right]\left(I_x^{\ell 1} + I_x^{u1}\right) + NM\left[\int_V \partial_y b_z\left(y - \frac{p}{4}, z_0\right) dV\right]\left(I_x^{\ell 2} + I_x^{u2}\right), \quad (22)$$

$$F_{zx} = NM\left[\int_V \partial_z b_z(y, z_0)\, dV\right]\left(I_x^{\ell 1} - I_x^{u1}\right) + NM\left[\int_V \partial_z b_z\left(y - \frac{p}{4}, z_0\right) dV\right]\left(I_x^{\ell 2} - I_x^{u2}\right), \quad (23)$$

$$\tau_x = NM\left[\int_V b_x(y, z_0)\, dV\right]\left(I_x^{\ell 1} - I_x^{u1}\right) + NM\left[\int_V b_x\left(y - \frac{p}{4}, z_0\right) dV\right]\left(I_x^{\ell 2} - I_x^{u2}\right), \quad (24)$$

$$k_y = -\frac{\partial F_y}{\partial y} = -NM\left[\int_V \partial_{yy} b_z(y, z_0)\, dV\right]\left(I_x^{\ell 1} + I_x^{u1}\right) - NM\left[\int_V \partial_{yy} b_z\left(y - \frac{p}{4}, z_0\right) dV\right]\left(I_x^{\ell 2} + I_x^{u2}\right). \quad (25)$$

It is seen that F_z can be obtained both by using the Y- traces and the X- traces. Thus, the total Z-force is given by

$$F_z = F_{zx} + F_{zy}. \quad (26)$$

Thus, for a desired Z-force F_z , the corresponding forces F_{zx} can be arbitrarily chosen, and F_{zy} would be obtained as $F_{zy} = F_z - F_{zx}$.

2.3. Approximating the magnet array with a point dipole and derivation of the resultant loads

Supplementary Fig. 3 shows that the magnetic fields per unit current $b_x(x, z_0)$ and $b_z(x, z_0)$ vary nearly sinusoidally along the X-axis. Thus,

$$b_x(x, z_0) \approx b_{1x}(z_0)\cos\left(\frac{2\pi x}{p}\right), \quad (27)$$

$$b_{zy}(x, z_0) \approx b_{1zy}(z_0)\sin\left(\frac{2\pi x}{p}\right). \quad (28)$$

Let the center of the magnet be assumed to be at the position (x_0, y_0, z_0) with respect to the origin in Supplementary Fig. (2). Neglecting the variation in magnetic field across the thickness of the magnet and considering the average magnetic field across to be the same as that at the levitation plane, then the expression for the force F_x can be simplified to be

$$F_x = NMb_{1zy}(z_0)t\frac{2\pi}{p}\left[\int_{x_0-\frac{p}{2}}^{x_0+\frac{p}{2}} L(x - x_0)\cos\left(\frac{2\pi x}{p}\right) dx\right] I_y^{\ell 1}. \quad (29)$$

Transforming the coordinates as $\theta = 2\pi(x - x_0)/p$, F_x can be written as

$$F_x = NMb_{1zy}(z_0)t\left[\int_{-\pi}^{\pi} L(p\theta/2\pi)\cos(\theta + \theta_0)\, d\theta\right] I_y^{\ell 1}. \quad (30)$$

Denoting $L_{11} = \int_{-\pi}^{\pi} L(p\theta/2\pi)\cos\theta\, d\theta$ and $L_{12} = \int_{-\pi}^{\pi} L(p\theta/2\pi)\sin\theta\, d\theta$, F_x can be written as

$$F_x = NMb_{1zy}(z_0)tL_1\cos(\theta_1 + \theta_0)\, I_y^{\ell 1}, \quad (31)$$

where, L_1, θ_1 and θ_0 are given by

$$L_1 = \sqrt{L_{11}^2 + L_{12}^2}, \quad (32)$$

$$\theta_1 = \tan^{-1}\frac{L_{12}}{L_{11}}, \quad (33)$$
$$\theta_0 = \frac{2\pi}{p}x_0.$$
Defining $m' = pNMtL_1/2\pi$, it is noted that the force experienced by the overall magnet is identical to the force experienced by a point dipole of magnitude m' placed at $x_1 = x_0 + \frac{p\theta_1}{2\pi}$.
For the specific case of a square-shaped magnet, $L(x - x_0)$ is given by
$$L(x - x_0) = p\left(1 - \frac{2|x - x_0|}{p}\right) \quad -p/2 \le x - x_0 \le p/2. \quad (34)$$
Using this in Eqns. (30) and (31), m' and x_1 are obtained to be
$$m' = \frac{4}{\pi^2}Nm, \quad (35)$$
$$x_1 = x_0\ . \quad (36)$$
This also applies to the computation of F_z and τ_y.

Thus, substituting this in all the equations, the force and currents are related as
$$F_x = \frac{m' b_{1zy}(z_0)2\pi}{p}\left(I_y^{\ell 1} + I_y^{u1}\right)\cos\left(\frac{2\pi x}{p}\right) + \frac{m' b_{1zy}(z_0)2\pi}{p}\left(I_y^{\ell 2} + I_y^{u2}\right)\sin\left(\frac{2\pi x}{p}\right), \quad (37)$$
$$k_x = \frac{m' b_{1zy}(z_0)4\pi^2}{p^2}\left(I_y^{\ell 1} + I_y^{u1}\right)\sin\left(\frac{2\pi x}{p}\right) - \frac{m' b_{1zy}(z_0)4\pi^2}{p^2}\left(I_y^{\ell 2} + I_y^{u2}\right)\cos\left(\frac{2\pi x}{p}\right). \quad (38)$$
Equations (37) and (38) can be rearranged as
$$\begin{bmatrix} \frac{p}{m' b_{1zy}(z_0)2\pi}F_x \\ -\frac{p^2}{m' b_{1zy}(z_0)4\pi^2}k_x \end{bmatrix} = \begin{bmatrix} \cos\left(\frac{2\pi x}{p}\right) & \sin\left(\frac{2\pi x}{p}\right) \\ -\sin\left(\frac{2\pi x}{p}\right) & \cos\left(\frac{2\pi x}{p}\right) \end{bmatrix}\begin{bmatrix} I_y^{\ell 1} + I_y^{u1} \\ I_y^{\ell 2} + I_y^{u2} \end{bmatrix}. \quad (39)$$
Similarly, it is noted that F_{zy} is given by
$$F_{zy} = NM\left[\int_V \partial_z b_z(x, z_0)\, dV\right]\left(I_y^{\ell 1} - I_y^{u1}\right) + NM\left[\int_V \partial_z b_z\left(x - \frac{p}{4}, z_0\right) dV\right]\left(I_y^{\ell 2} - I_y^{u2}\right). \quad (40)$$

By employing the fact that $\nabla \cdot \mathbf{B} = 0$, it can be concluded that $\partial_z b_z(x, z_0) = -\partial_x b_x(x, z_0)$. Utilizing the expression for $b_x(x, z_0)$ given in Eqn. (27), the force F_{zy} and τ_y can be related to the currents as
$$\begin{bmatrix} \frac{1}{m' b_{1x}(z_0)}\tau_y \\ -\frac{p}{m' b_{1x}(z_0)2\pi}F_{zy} \end{bmatrix} = \begin{bmatrix} \cos\left(\frac{2\pi x}{p}\right) & \sin\left(\frac{2\pi x}{p}\right) \\ -\sin\left(\frac{2\pi x}{p}\right) & \cos\left(\frac{2\pi x}{p}\right) \end{bmatrix}\begin{bmatrix} I_y^{\ell 1} - I_y^{u1} \\ I_y^{\ell 2} - I_y^{u2} \end{bmatrix}. \quad (41)$$
Finally, defining $\mathbf{I_y} = \left[I_y^{l2} - I_y^{u2} \quad I_y^{l1} - I_y^{u1} \quad I_y^{l1} + I_y^{u1} \quad I_y^{l2} + I_y^{u2}\right]^T$ and $\mathbf{F_{Iy}} = \frac{1}{m'}\left[-\frac{p}{b_{1x}2\pi}F_{zy} \quad \frac{1}{b_{1x}}\tau_y \quad \frac{p}{b_{1zy}2\pi}F_x \quad -\frac{p^2}{b_{1zy}4\pi^2}k_x\right]^T$, and $\mathbf{R}\left(\frac{2\pi x}{p}\right) = \begin{bmatrix} \cos\left(\frac{2\pi x}{p}\right) & -\sin\left(\frac{2\pi x}{p}\right) \\ \sin\left(\frac{2\pi x}{p}\right) & \cos\left(\frac{2\pi x}{p}\right) \end{bmatrix}$, it is noted that
$$\mathbf{F_{Iy}} = \begin{bmatrix} \mathbf{R}\left(\frac{2\pi x}{p}\right) & \mathbf{0}_{2\times 2} \\ \mathbf{0}_{2\times 2} & \mathbf{R}\left(-\frac{2\pi x}{p}\right) \end{bmatrix}\mathbf{I_y}. \quad (42)$$

It is worth noting that $\mathbf{R}\left(\frac{2\pi x}{p}\right)$ is a rotation matrix. Thus, $\mathbf{R^{-1}}\left(\frac{2\pi x}{p}\right) = \mathbf{R}\left(-\frac{2\pi x}{p}\right)$. Hence the current vector $\mathbf{I_y}$ necessary to achieve a specified $\mathbf{F_{Iy}}$ is given by

$$\mathbf{I_y} = \begin{bmatrix} \mathbf{R}\left(-\frac{2\pi x}{p}\right) & \mathbf{0}_{2\times 2} \\ \mathbf{0}_{2\times 2} & \mathbf{R}\left(\frac{2\pi x}{p}\right) \end{bmatrix} \mathbf{F_{Iy}}. \tag{43}$$

In a similar manner, It can be shown that $\boldsymbol{I_x} = [I_x^{l2} - I_x^{u2} \quad I_x^{l1} - I_x^{u1} \quad I_x^{l1} + I_x^{u1} \quad I_x^{l2} + I_x^{u2}]^T$ and $\mathbf{F_{Ix}} = \frac{1}{m'}\left[-\frac{p}{b_{1y}2\pi}F_{zx} \quad \frac{1}{b_{1y}}\tau_x \quad \frac{p}{b_{1zx}2\pi}F_y \quad -\frac{p^2}{b_{1zx}4\pi^2}k_y\right]^T$ are related as

$$\mathbf{I_x} = \begin{bmatrix} \mathbf{R}\left(-\frac{2\pi y}{p}\right) & \mathbf{0}_{2\times 2} \\ \mathbf{0}_{2\times 2} & \mathbf{R}\left(\frac{2\pi y}{p}\right) \end{bmatrix} \mathbf{F_{Ix}}. \tag{44}$$

Thus, by using Eqns. (43) and (44), it is possible to generate current vectors $\mathbf{I_y}, \mathbf{I_x}$ to generate any specified load vectors $\mathbf{F_{Iy}}, \mathbf{F_{Ix}}$.

To verify the point-dipole approximation in Eqn. (35), the Z-force of interaction F_{zy} between the magnet array and a single Y-trace on the bottom PCB was experimentally measured as function of the X-position above the trace by mounting the magnet array rigidly on a cantilever beam, and measuring the deformation of the cantilever beam due to the interaction force. The beam was chosen to be stiff enough that its deformation did not substantially change the height z of the magnet above the trace. Supplementary Fig. 4 (a) shows the schematic of the experimental set-up employed for the measurement. The cantilever was mounted on a 3-axis motorized stage to translate it along the X-axis. A side microscope was used for positioning the magnet array at a known height z above the Y-trace, while the deflection of the cantilever was measured using the top microscope. The measured displacement was multiplied with the stiffness of the cantilever to obtain the Z-force F_{zy}. Supplementary Fig. 4(b) plots the normalized Z-force F_{zy}/F_0 as a function of X-position of the magnet array and compares it with the calculated value. Here F_0 represents the characteristic force given by $F_0 = \mu_0 N m I_y^{l1}/2\pi p^2$. The employed stainless steel cantilever's length, width, and thickness are 100 *mm*, 25 *mm*, and 0.9 *mm*, respectively. The Young's modulus of the stainless steel is 209 *GPa*. It is seen that the force is nearly sinusoidal, and the amplitude of the sinusoid agrees with the calculated value to within 20%. The difference between the two may be attributed to uncertainty in the stiffness of the cantilever due to corresponding uncertainty of its dimensions.

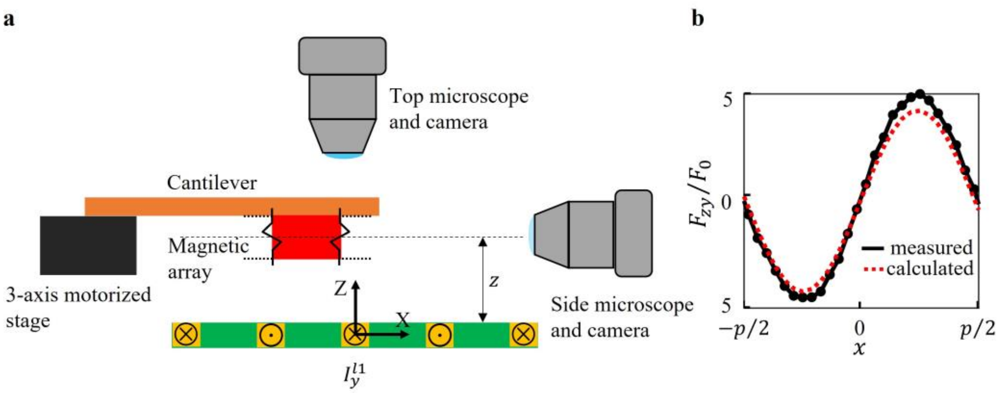

Supplementary Fig. 4. **Schematic of the experimental set-up used for force measurement and the plot comparing the experimentally measured force with the calculated force.** (a) Schematic showing the experimental set-up employed for the measurement of Z-force acting on a magnet array, (b) Plots showing the measured and analytically calculated Z-force F_{zy}/F_0 acting on the 3×3 magnet array as a function of the X-position of the magnet. The Z-position of the magnet was kept constant at 800 µm. The stiffness of the employed cantilever was 943 *N/m.* The value of N, m, p and I_y^{l1} are $9, 0.0012\ Am^2, 2.54\ \times 10^{-3}\ m$ and 1 *A* respectively.

2.4. Maximum loads that can be applied by the actuator

The maximum loads that can be applied is ultimately limited by the magnitude of the maximum current I_{max} that can be passed through the traces, which in turn, is chiefly limited by joule heating caused by passing higher currents. While the maximum values can be obtained by using the exact relationships between loads and current, given in Eqns. (14)-(25), nearly identical results can be obtained under the assumption that $b_x(x, z_0)$ and $b_z(x, z_0)$ varies sinusoidally in the levitation plane. In this case, Eqns. (37)-(42) can be employed to obtain these maximum values.

Thus, by substituting $|I_y^{\ell 1}| = |I_y^{u1}| = |I_y^{\ell 2}| = |I_y^{u2}| = I_{max}$ in Eqn. (37), the maximum X-force $F_{x,max}$ is obtained. By symmetry, this would be the same as the maximum Y-force $F_{y,max}$ as well and is given by

$$F_{x,max} = F_{y,max} = \frac{4\sqrt{2}\pi}{p} m' b_{1zy}(z_0) I_{max}. \quad (45)$$

Since the Z-force can be generated either by using Y-traces or by using X-traces, the maximum Z-force is given by

$$F_{z,max} = \frac{8\sqrt{2}\pi}{p} m' b_{1x}(z_0) I_{max} \ . \quad (46)$$

Similarly, the maximum torque is given by

$$\tau_{x,max} = \tau_{y,max} = \frac{8\sqrt{2}}{\pi^2} m' b_{1zy}(z_0) I_{max.} \quad (47)$$

It is useful to express the maximum forces as a multiple of the total weight $W = \rho V g$ of a magent of volume V and density ρ. Noting that $m' = 4Nm/\pi^2$, and $m = MV$, the expression for $F_{x,max}/W$ and $F_{z,max}/W$ are given by

$$\frac{F_{x,max}}{W} = \frac{16\sqrt{2}}{\pi} \frac{M b_{1zy}(z_0)}{p\rho g} I_{max.} \quad (48)$$

$$\frac{F_{z,max}}{W} = \frac{32\sqrt{2}}{\pi}\frac{Mb_{1x}(z_0)}{p\rho g}I_{max.} \tag{49}$$

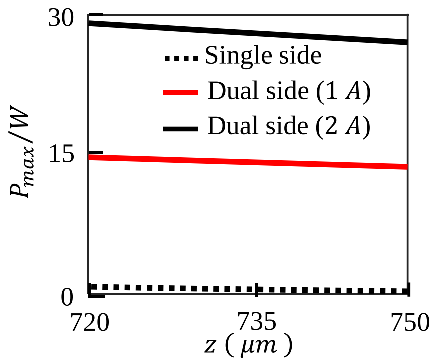

Supplementary Fig. 5. **Comparison of the payload carrying capacities of single and dual side actuators:** Plot showing the normalized payload carrying capacity P_{max}/W of the single and dual sided actuator. The plots in solid red line and solid black line are obtained for I_{max} to be 1 A and 2 A respectively for the case of dual-sided actuation.

Eqn. (49) also helps to obtain the payload carrying capacity P_{max} for dual-sided actuation by noting that $F_{z,max} = P_{max} + W$. If a lesser levitation height z is chosen, then b_{1x} would be correspondingly higher and the payload carrying capacity would also be higher.
In contrast, for single-sided actuation, it is not possible to employ actuation current to compensate for the weight of the payload because of the cross-coupling between the Z-force and the trap stiffness along the in-plane axes: such a vertical force would destabilize the trap along the in-plane axes. Thus, the load carrying capacity P_{max} at any levitation height z would be $P_{max} = F_d(z) - W$. For the parameters of the set-up chosen in the paper, Supplementary Fig. 5 plots the maximum payload carrying capacity, normalized with respect to W, for the dual-sided actuator and compares the same with that of single-sided actuation. It is seen that the load carrying capacity for dual-sided actuation is approximately 50 times the capacity of single-sided actuation for the case $I_{max} = 2A$.

2.5. Relationship between the stiffness along X-, Y- and Z-axes

If $\mathbf{B} = [B_x \quad B_y \quad B_z]^T$ represents the magnetic field set-up by all the eight traces combined, then, the stiffness along X-, Y- and Z-axes are given by

$$k_x = -\frac{\partial F_x}{\partial x} = -NM\int_V \partial_{xx}B_z dV, \tag{50}$$

$$k_y = -\frac{\partial F_y}{\partial x} = -NM\int_V \partial_{yy}B_z dV, \tag{51}$$

$$k_z = -\frac{\partial F_d}{\partial z} - \frac{\partial F_z}{\partial z} = k_d - NM\int_V \partial_{zz}B_z dV. \tag{52}$$

Where k_d represents the diamagnetic stiffness and is given by $k_d = -\frac{\partial F_d}{\partial z}$.

Thus,

$$k_x + k_y + k_z = k_d - NM\int_V \nabla^2 B_z dV\ . \tag{53}$$

Since $\nabla \cdot \mathbf{B} = 0$, it is noted that $\partial_z(\nabla \cdot \mathbf{B}) = 0$, or equivalently,

$$\partial_{zx}B_x + \partial_{zy}B_y + \partial_{zz}B_z = \partial_x(\partial_z B_x) + \partial_y(\partial_z B_y) + \partial_{zz}B_z = 0. \tag{54}$$

Given that $\nabla \times \mathbf{B} = \mathbf{0}$, it can be noted that $\partial_z B_x = \partial_x B_z$ and $\partial_z B_y = \partial_y B_z$. Thus, Eqn. (54) reduces to

$$\partial_{xx}B_z + \partial_{yy}B_z + \partial_{zz}B_z = \nabla^2 B_z = 0. \tag{55}$$

Thus, using Eqn. (55) in Eqn. (53), it is seen that

$$k_x + k_y + k_z = k_d. \tag{56}$$

2.6 Workspace of the actuator

This section discusses the workspace of the actuator. The area of the workspace along X- and Y-axes is determined by the area of the PCBs covered by the meanders along each axis. The workspace along the Z-axis is limited from below by the possibility of contact with the graphite plate, while it is limited from above by the height at which the magnet array becomes unstable. The workspace below the levitation plane is equal to the air gap, i.e., $z_0 - t_d - t/2$. Numerically, this is $51\ \mu m$. Above the levitation plane, it is limited by the height z_1 at which $k_z = 0$. Beyond this height, the magnet array would be unstable since k_z would be negative. Since $k_z = k_d(z) - (k_x + k_y)$, the height z_1 is obtained from the equation

$$k_d(z_1) = k_x + k_y. \tag{57}$$

Thus, the total workspace along the Z-axis is given to be

$$z_w = z_1 - t_d - t/2. \tag{58}$$

For the case that $k_x = k_y = k_d/3$, z_1 is determined to be $z_1 = 801\ \mu m$ or equivalently, about $50\ \mu m$ above the levitation plane.

The height of the workspace along the Z-axis, is therefore $z_w = 101\ \mu m$. It is seen that the height of the workspace z_w is much lesser than the gap between the PCBs, viz., $1502 \mu m$. Thus, the variation in electromagnetic stiffness along the X- and Y-axes is also small within this range, namely, about 1.4%.

The achievable angular range for rotations θ_x, θ_y about X- and Y-axes respectively are also limited by the possibility of contact with the graphite plate. Since the lateral extent of the magnet array is much larger than the gap between the graphite plate and the array throughout the workspace of the actuator, θ_x, θ_y would be correspondingly small. If the center of the array is at a height z above the bottom PCB and if x_f and y_f represent the X- and Y-coordinates of the point on the magnet array which is furthermost from the axis of rotation, the height of this point above the bottom PCB is given by

$$z_f = z - \theta_x y_f + \theta_y x_f. \tag{59}$$

The rotation range is limited by the requirement that $z_f \geq t_d + t/2$, in order to avoid contact with the graphite plate.

If the magnet array is a $n \times n$ square, for rotations purely about X- or Y-axes, the angular range at the specified z is given to be $\theta_{max} = 2(z - t_d - t/2)/np$. θ_{max} assumes its maximum value at the upper limit of the workspace, i.e., $z = z_1$. For a 3× 3 magnet array, this is about $26.5\ mrad$.

Supplementary Note 3: Drive configurations and dynamic modeling of the multi-zone nanopositioner

3.1 Drive configuration to achieve multi-degree of freedom positioning

This section describes the procedure and the currents necessary to achieve simultaneous positioning of the nanopositioner along all the six degrees of freedom. It is noted that within the pitch p of a single meander, the loads are uniquely related to the actuation currents and that this repeats itself with a periodicity of p along the X- and Y- axes. This fact is employed to obtain the drive configurations to achieve the desired multi degree-of-freedom position in three steps: In the first step, the specified linear displacement along X- and Y-axes x_s, y_s and angular displacement about the Z-axis θ_{zs} are achieved. In the second step, the loads that need to be applied to achieve the specified Z- displacement z_s and angular displacements about X- and Y-axes θ_{xs}, θ_{ys} are obtained. In the third step, the X- and Y-stiffnesses k_{xs}, k_{ys} are also specified and the actuation currents necessary to apply the loads and achieve the stiffnesses at the specified position are obtained using Eqns. (43-44) derived in the Supplementary note 2.3. These steps are elaborated below.

In the first step, the desired linear displacement along X- and Y-axes x_s, y_s and angular displacement about the Z-axis θ_{zs} are achieved by suitably shifting the equilibrium points of the magnet array in each of the zones of the actuator. To achieve linear displacements x_s, y_s the equilibrium points in all of the zones are shifted along the X- and Y-axes with speeds v_x, v_y respectively, and for durations $t_x = x_s/v_x$ and $t_y = y_s/v_y$ respectively. This is achieved by applying the current waveforms:

$$I_y^{l1} = I_y^{u1} = I_{0x} sin\left(\frac{2\pi}{p} v_x t\right), \quad (60)$$

$$I_y^{l2} = I_y^{u2} = I_{0x} cos\left(\frac{2\pi}{p} v_x t\right), \quad (61)$$

$$I_x^{l1} = I_x^{u1} = I_{0y} sin\left(\frac{2\pi}{p} v_y t\right), \quad (62)$$

$$I_x^{l2} = I_x^{u2} = I_{0y} cos\left(\frac{2\pi}{p} v_y t\right). \quad (63)$$

In Eqns. (60)-(63), I_{0x}, I_{0y} are chosen to be $I_{0x} = \frac{p^2}{8\sqrt{2}\pi^2 m' b_{1zy}} k_x$ and $I_{0y} = \frac{p^2}{8\sqrt{2}\pi^2 m' b_{1zy}} k_y$. This ensures that the specified trap stiffness is maintained during the displacements.

To achieve angular displacement θ_{zs}, the equilibrium points in diagonally opposite zones are shifted by equal and opposite amounts tangential to the magnet array. For small θ_{zs}, this is given by $l_a \theta_{zs}$ where l_a represents the distance of the center of the arm from the axis of rotation . The X- and Y- waveforms are similar to those described in Eqns. (60)-(63). The sign of X- and Y- displacements in each zone is chosen to ensure that the magnet array moves tangential to the center of rotation and ensures rotation of the magnet array about the Z-axis by the desired amount. For large rotations, two issues are introduced from the view-point of analysis: first, the locations of the centers of the magnets will no longer be at the desired pitch along X- and Y-axes, and second, the in-plane rotation of the magnets changes the resulting point dipole m'. While the drive configuration for large angle rotation can also be derived for this case, a more appropriate arrangement of traces to rotate by large angles would be to employ traces patterned in the radial and azimuthal directions on the PCB.

In the second step, the specified position of the center of the actuator along the Z-axis is given to be z_s and desired orientations about X- and Y-axes are given to be θ_{xs} and θ_{ys} respectively. If

x_i and y_i represent the X- and Y-coordinates of the center of the ith magnet in the magnet array, the height of the magnet above the bottom PCB is given by

$$z_i = z_s - \theta_{xs} y_i + \theta_{ys} x_i. \quad (64)$$

The Z-force necessary to position the magnet array in this configuration is given by $[W - \sum_i F_d(z_i)]$. Thus, the load on each arm would be 1/4th of this value. Further in each arm, this force is applied jointly by the X- and Y- traces. Thus, both X- and Y- traces has to apply force F_{zs} given by:

$$F_{zs} = 0.125[W - \sum_i F_d(z_i)]. \quad (65)$$

The necessary torque about X- and Y-axes on each arm is given by $\boldsymbol{\tau}_\mathbf{s} = 0.25 \sum_i \mathbf{r_i} \times \left(\mathbf{F_d}(z_i)\right)$, where $\mathbf{r_i} = [x_i \; y_i \; 0]^T$ and $\mathbf{F_d}(z_i) = [0 \; 0 \; F_d(z_i)]^T$. The resulting X-, Y-components of $\boldsymbol{\tau}_\mathbf{s}$ are given by

$$\tau_{xs} = 0.25 \sum_i F_d(z_i) y_i, \quad (66)$$

$$\tau_{ys} = -0.25 \sum_i F_d(z_i) x_i. \quad (67)$$

Eqns. (64)-(67) specify all the loads necessary for achieving the desired orientation and Z-position.

In the third step, the necessary currents in each of the zones are obtained by using Eqns. (43)-(44). If x_j, y_j represent the X- and Y-displacements of the equilibrium in zone j of the actuator ($j = 1,2,3,4$), the resulting currents for this zone are given by

$$\begin{bmatrix} I_y^{l1} \\ I_y^{l2} \\ I_y^{u1} \\ I_y^{u2} \end{bmatrix} = \begin{bmatrix} A_y^{l1}(x_j) & B_y^{l1}(x_j) & C_y^{l1}(x_j) \\ A_y^{l2}(x_j) & B_y^{l2}(x_j) & C_y^{l2}(x_j) \\ A_y^{u1}(x_j) & B_y^{u1}(x_j) & C_y^{u1}(x_j) \\ A_y^{u2}(x_j) & B_y^{u2}(x_j) & C_y^{u2}(x_j) \end{bmatrix} \begin{bmatrix} \tau_{ys} \\ F_{zs} \\ k_{xs} \end{bmatrix} \quad (68)$$

$$\begin{bmatrix} I_x^{l1} \\ I_x^{l2} \\ I_x^{u1} \\ I_x^{u2} \end{bmatrix} = \begin{bmatrix} A_x^{l1}(y_j) & B_x^{l1}(y_j) & C_x^{l1}(y_j) \\ A_x^{l2}(y_j) & B_x^{l2}(y_j) & C_x^{l2}(y_j) \\ A_x^{u1}(y_j) & B_x^{u1}(y_j) & C_x^{u1}(y_j) \\ A_x^{u2}(y_j) & B_x^{u2}(y_j) & C_x^{u2}(y_j) \end{bmatrix} \begin{bmatrix} \tau_{xs} \\ F_{zs} \\ k_{ys} \end{bmatrix}, \quad (69)$$

where, the coefficients of the matrices are given to be

$$A_y^{l1}(x_j) = -A_y^{u1}(x_j) = \frac{1}{2m' b_{1x}} cos\left(\frac{2\pi x_j}{p}\right), A_x^{l1}(y_j) = -A_x^{u1}(y_j) = \frac{1}{2m' b_{1x}} cos\left(\frac{2\pi y_j}{p}\right),$$

$$A_y^{l2}(x_j) = -A_y^{u2}(x_j) = \frac{1}{2m' b_{1x}} sin\left(\frac{2\pi x_j}{p}\right), A_x^{l2}(y_j) = -A_x^{u2}(y_j) = \frac{1}{2m' b_{1x}} sin\left(\frac{2\pi y_j}{p}\right),$$

$$B_y^{l1}(x_j) = -B_y^{u1}(x_j) = \frac{p}{4\pi m' b_{1x}} sin\left(\frac{2\pi x_j}{p}\right), B_x^{l1}(y_j) = -B_x^{u1}(y_j) = \frac{p}{4\pi m' b_{1x}} sin\left(\frac{2\pi y_j}{p}\right)$$

$$B_y^{l2}(x_j) = -B_y^{u2}(x_j) = -\frac{p}{4\pi m' b_{1x}} cos\left(\frac{2\pi x_j}{p}\right), B_y^{l2}(y_j) = -B_y^{u2}(y_j) = -\frac{p}{4\pi m' b_{1x}} cos\left(\frac{2\pi y_j}{p}\right)$$

$$C_y^{l1}(x_j) = C_y^{u1}(x_j) = -\frac{p^2}{8\pi^2 m' b_{1zy}} sin\left(\frac{2\pi x_j}{p}\right), C_y^{l1}(y_j) = C_y^{u1}(y_j) = -\frac{p^2}{8\pi^2 m' b_{1zy}} sin\left(\frac{2\pi y_j}{p}\right)$$

$$C_y^{l2}(x_j) = C_y^{u2}(x_j) = \frac{p^2}{8\pi^2 m' b_{1zy}} cos\left(\frac{2\pi x_j}{p}\right), C_y^{l2}(y_j) = C_y^{u2}(y_j) = \frac{p^2}{8\pi^2 m' b_{1zy}} cos\left(\frac{2\pi y_j}{p}\right).$$

It is noted that , $x_j = x_s - \frac{l_a\theta_{zs}}{\sqrt{2}}$ for zone numbers $j = 1,2$ and $x_j = x_s + \frac{l_a\theta_{zs}}{\sqrt{2}}$ for zone numbers $j = 3,4$. Likewise $y_j = y_s + \frac{l_a\theta_{zs}}{\sqrt{2}}$ for zone numbers $j = 1,4$ and $y_j = y_s - \frac{l_a\theta_{zs}}{\sqrt{2}}$ for zone numbers $j = 2,3$.

Eqn. (68) and (69) specify the drive configuration of the nanopositioner to simultaneously displace the positioner by the specified amount along all the six degrees of freedom. These equations can be employed to compute the maximum value of currents for the specified loads and stiffnesses. Since I_y^{l1} is given by $\frac{\tau_{ys}}{2m'b_{1x}}\cos\left(\frac{2\pi x}{p}\right) + \left(\frac{pF_{zs}}{4\pi m'b_{1x}} - \frac{p^2k_{xs}}{8\pi^2m'b_{1zy}}\right)\sin\left(\frac{2\pi x}{p}\right)$, its maximum value is $\sqrt{\left(\frac{\tau_{ys}}{2m'b_{1x}}\right)^2 + \left(\frac{pF_{zs}}{4\pi m'b_{1x}} - \frac{p^2k_{xs}}{8\pi^2m'b_{1zy}}\right)^2}$. Following this procedure, the maximum value of all the currents is given by

$$I_{ymax}^{l1} = I_{ymax}^{l2} = \sqrt{\left(\frac{\tau_{ys}}{2m'b_{1x}}\right)^2 + \left(\frac{pF_{zs}}{4\pi m'b_{1x}} - \frac{p^2k_{xs}}{8\pi^2m'b_{1zy}}\right)^2}, \tag{70}$$

$$I_{ymax}^{u1} = I_{ymax}^{u2} = \sqrt{\left(\frac{\tau_{ys}}{2m'b_{1x}}\right)^2 + \left(\frac{pF_{zs}}{4\pi m'b_{1x}} + \frac{p^2k_{xs}}{8\pi^2m'b_{1zy}}\right)^2}, \tag{71}$$

$$I_{xmax}^{l1} = I_{xmax}^{l2} = \sqrt{\left(\frac{\tau_{xs}}{2m'b_{1x}}\right)^2 + \left(\frac{pF_{zs}}{4\pi m'b_{1x}} - \frac{p^2k_{ys}}{8\pi^2m'b_{1zy}}\right)^2}, \tag{72}$$

$$I_{xmax}^{u1} = I_{xmax}^{u2} = \sqrt{\left(\frac{\tau_{xs}}{2m'b_{1x}}\right)^2 + \left(\frac{pF_{zs}}{4\pi m'b_{1x}} + \frac{p^2k_{ys}}{8\pi^2m'b_{1zy}}\right)^2}. \tag{73}$$

Since the loads in Eqns. (70)-(73) are functions of the specified displacements along the different degrees of freedom, these equations can be employed to obtain the workspace as determined by the current limit I_{max}. However, upon computing the currents within the workspace determined by spatial contraints and instability, as discussed in Supplementary note 2.6, the maximum value of current is found to be 245 mA, which is much less than $I_{max} = 2A$. Thus, this indicates that for the specified I_{max}, workspace is determined by the factors described in Supplementary note 2.6 and not by the current limit.

3.2 Dynamic modeling and characterization of the nanopositioner

In equilibrium, each magnet in the magnet array is electromagnetically trapped with trap stiffness being k_x, k_y and k_z along X-, Y- and Z-axes respectively. Thus, for small displacements away from equilibrium, the dynamic behavior would be that of a mass-spring-damper system, where the damping arises due to the surrounding air. If , m_a represents the mass of the overall magnet array, and b_x represents the damping coefficient, the dynamic model along the X-axis would be

$$m_a\ddot{x} + b_x\dot{x} + k_x x = f_x, \tag{74}$$

where, f_x represents the force along the X-axis. Similar equations would be valid for motion along Y- and Z-axes as well.

A linear second-order system model would also be valid for angular dynamics about X-, Y- and Z-axis as well, provided the angular displacements are small. The model along the X-axis is given to be

$$I_{xx}\ddot{\theta}_x + b_\theta \dot{\theta}_x + k_{\theta x}\theta_x = \tau_x, \quad (75)$$

where, I_{xx} represents the moment of inertia of the magnet array about the X-axis, b_θ represents the angular damping coefficient and $k_{\theta x}$ represents the angular stiffness about the X-axis, while τ_x represents the torque. Similar equations would be valid for small rotations about the Y- and Z-axes as well.

The angular stiffness $k_{\theta x}$ can be obtained by noting that if x_i represents the offset of the center of the ith magnet in the magnet array, a small angular displacement θ_x results in a Z-displacement of $\theta_x x_i$ and causes the magnet array to experience a restoring force of $-k_z\theta_x x_i$. The moment of this force about the X-axis is given by $-k_z\theta_x x_i^2$. Thus, the torsional stiffness is given to be

$$k_{\theta x} = k_z \sum_i x_i^2. \quad (76)$$

Likewise, the angular stiffness about Y-axis is given by,

$$k_{\theta y} = k_z \sum_i y_i^2,$$

while the Z-angular stiffness is obtained to be

$$k_{\theta z} = k_x \sum_i y_i^2 + k_y \sum_i x_i^2. \quad (77)$$

These analyses reveal that the dynamic model of the actuator for small displacements is that of a second order system along all its degrees of freedom. This was validated experimentally from the step responses obtained along the linear and angular channels and the bandwidth of the nanopositioner along the X-, and Z- linear channels and θ_x, θ_z angular channels was evaluated from these responses. In the experiments, the X- and Y-stiffnesses were chosen to be the same. Thus, by symmetry, the linear dynamic responses along X- and Y-axes would be identical as also the angular dynamic responses about X- and Y-axes.

Supplementary Fig. 6 (a)-(d) plots the step responses along the four axes and also the fitted second order models, whose coefficients were chosen to match the experimental step responses. The insets in each figure show the responses in the vicinity of time $t = 0$. It is seen from the figures that the step responses of the second order models match the experimental responses well. The natural frequencies and the damping factors for translation along X-, Y- and Z-axes are $\omega_{nx} = \omega_{ny} = 130.8\, rad/s, \omega_{nz} = 140.7\, rad/s$, $\xi_x = \xi_y = 0.005$ and $\xi_z = 0.10$. Similarly the natural frequencies and damping factors for rotations about X- , Y- and Z-axes are $\omega_{n\theta_x} = \omega_{n\theta_y} = 110.8\, rad/s, \omega_{n\theta_z} = 89.7\, rad/s, \xi_{\theta_x} = \xi_{\theta_y} = 0.12$ and $\xi_{\theta_z} = 0.007$. The damping factor is seen to be larger and hence the settling time is smaller for actuation resulting in out-of-plane displacements compared to actuation resulting in in-plane displacements. This may be attributed to the squeeze-film damping effect arising due to the narrow air gap between the graphite plate and the magnet array.

Supplementary Fig. 7 (a)-(d) plot the magnitude responses of the fitted second-order models along each axis and shows that the open-loop bandwidth is $\omega_{bx} = \omega_{by} = 131\, rad/s$, $\omega_{bz} = 153\, rad/s$, $\omega_{b\theta_z} = 90\, rad/s$ and $\omega_{b\theta_x} = \omega_{b\theta_y} = 121\, rad/s$.

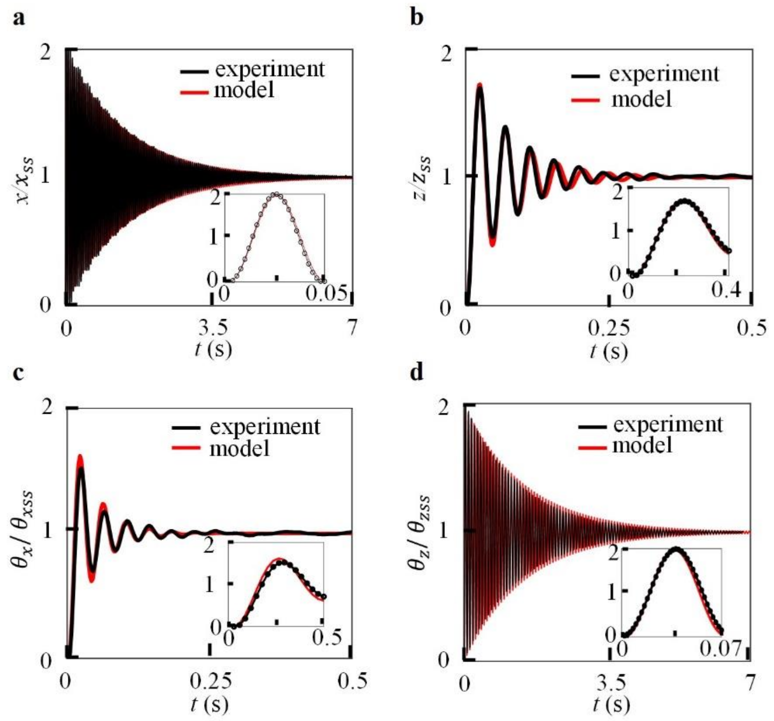

Supplementary Fig. 6. **Step responses of the positioner along the linear and angular degrees-of-freedom:** Plots showing the step response along the four axes: (a) X- axis, (b) Z-axis, (c) θ_z- axis and (d) θ_x- axis. The measurements have been normalized with respect to their steady-state values $x_{ss}, z_{ss}, \theta_{xss}$ and θ_{zss} respectively. Initially the currents through all the traces of the multi-zone positioner were maintained constant at 200 *mA*. Next the currents through appropriate traces were increased by 10 mA to achieve the required translational and rotational step responses.

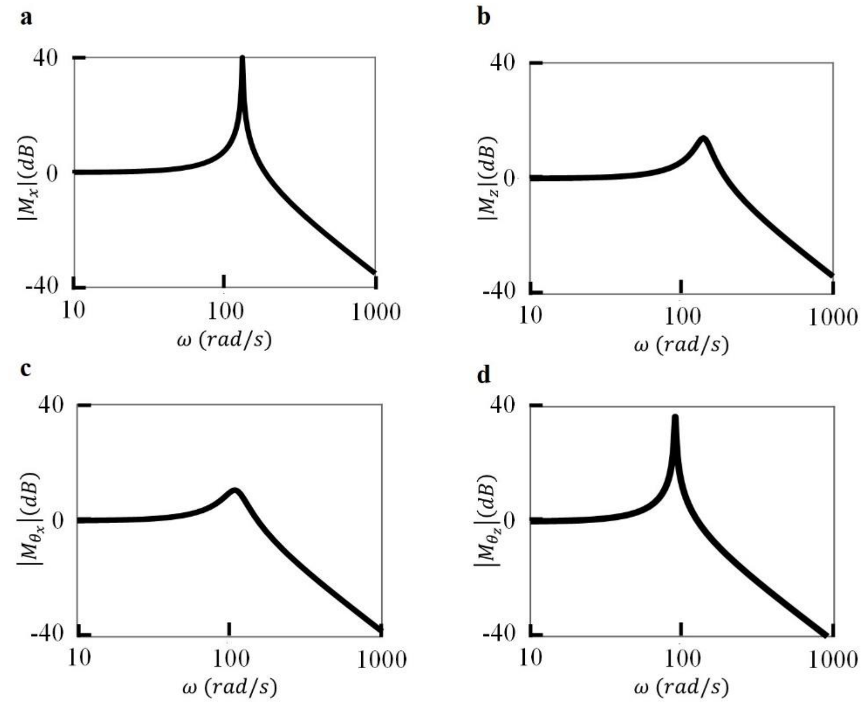

Supplementary Fig. 7. **Magnitude frequency responses of the positioner along the linear and angular degrees-of-freedom:** Plots showing the magnitude response of the fitted second order model along the four axes : (a) X- axis, (b) Z-axis, (c) θ_x- axis and (d) θ_z- axis.
