# Supplementary material for: Diamagnetically levitated nanopositioners with large-range and multiple degrees of freedom
Source: Nat Commun. 2022 Jun 9;13:3334. doi: 10.1038/s41467-022-31046-4 (PMC9184538; doi:10.1038/s41467-022-31046-4)
Supplement: Supplementary file 3 — Description of Additional Supplementary Files [file 41467_2022_31046_MOESM3_ESM.pdf]

Description of Additional Supplementary Files

File Name: Supplementary Movie 1
Description: Multi-zone positioner

File Name: Supplementary Movie 2
Description: Flexure-based positioner
